# Comparative Study of Post-Surgical Outcomes in Pain, Disability, and Health-Related Quality of Life for Adult Spinal Deformity in Patients Aged above and below 75 Years

**DOI:** 10.3390/life13122329

**Published:** 2023-12-12

**Authors:** Yeonsu Park, Jiyoon Kim, Ho-Joong Kim, Seungtak Oh, Joon-Hee Park, Daechul Shim, Jin-Ho Park

**Affiliations:** 1 College of Medicine, Seoul National University, 103, Daehak-ro, Jongno-gu, Seoul 03080, Republic of Korea; 2 Spine Center and Department of Orthopedic Surgery, Seoul National University College of Medicine and Seoul National University Bundang Hospital, 166 gumiro, Bundang-gu, Sungnam-si 13620, Republic of Korea; 54551@snubh.org; 3 Department of Anesthesiology and Pain Medicine, Kangdong Sacred Heart Hospital, Hallym University College of Medicine, 18, Cheonho-daero 173-gil, Gangdong-gu, Seoul 05355, Republic of Korea; joonheepark93@gmail.com (J.-H.P.); daechuba@gmail.com (D.S.); 4 Department of Orthopedic Surgery, Kangdong Sacred Heart Hospital, Hallym University College of Medicine, 18, Cheonho-daero 173-gil, Gangdong-gu, Seoul 05335, Republic of Korea

**Keywords:** adult spinal deformity, pain, disability, quality of life, complication

## Abstract

(1) Background: Adult spinal deformity (ASD) surgery is known to improve clinical and radiological parameters; however, it may also cause more complications in elderly patients. The purpose of this study was to compare the outcomes of ASD surgery, specifically regarding pain, disability, and health-related quality of life (HRQOL) in patients aged 75 years and over and patients aged under 75 years; (2) Methods: A total of 151 patients who underwent ASD surgery between August 2014 and September 2020 were included. Patients were divided into two groups based on whether they are 75 years and over or under. Radiological parameters measured included sagittal vertical axis (SVA), pelvic tilt (PT), and pelvic incidence (PI)- lumbar lordosis (LL). Data were collected 3, 6, and 12 months after surgery; (3) Results: At 12 months postoperatively, visual analog scale (VAS) for low back pain (*p* = 0.342), Oswestry disability index (ODI) (*p* = 0.087), and EuroQol 5-Dimensions (EQ-5D) (*p* = 0.125) did not differ between patients under 75 years and those 75 and above 75 group. PT (*p* = 0.675), PI-LL (*p* = 0.948), and SVA (*p* = 0.108) did not differ significantly 12 months after surgery in the two groups. In the entire patient group, compared to preoperative data, significant improvements were demonstrated for clinical and radiological parameters 12 months after surgery (all *p* < 0.001). The rate of medical complications did not correlate with age, but the rates of proximal junctional kyphosis (PJK) and proximal junctional failure (PJF) did (*p* = 0.638, *p* < 0.001, and *p* = 0.001, respectively); (4) Conclusions: In terms of clinical and radiological improvements, ASD surgery should be considered for patients regardless of whether they are younger than or older than 75 years. The clinical and radiological improvements and the risk of complications and revision surgeries must be considered in ASD patients who are 75 years or older.

## 1. Introduction

Adult spinal deformity (ASD) is believed to be a result of accumulated spinal degenerative changes with aging and refers to deformities in spinal curvature or alignment affecting the axial, coronal, and sagittal planes [1,2]. Some studies have reported that chronic recurrent multifocal osteomyelitis may also contribute to deformities of the spine [3]. ASD is known to develop with age and is therefore one of the most prevalent diseases affecting elderly people. The World Health Organization report suggested that by 2030, one in six people will be aged 60 years or older, and the worldwide prevalence of ASD is increasing with our aging population. ASD is a condition that can cause serious pain and poor quality of life, and therefore, the correction is very important [4,5,6,7].

Adult spinal deformity frequently accompanies low back pain and sciatica which have significant impacts on patient quality of life [8]. Some studies have reported that exercise therapy, including Pilates, can be effective in treating spinal deformity, but spinal surgery is known to be an effective means of alleviating pain [9,10,11], and research conducted on patients above 65 years of age have shown significant post-operational improvements in health-related quality of life (HRQOL) [12,13,14].

In older age, spinal surgery is known to increase surgical morbidity and mortality [15,16,17]. Therefore, deciding to perform ASD surgery in elderly patients is not an easy choice, and the outcomes of the surgery must justify its risks. According to previous studies, ASD surgery in patients over 75 years old has been reported to be effective in reducing pain and disability compared to non-surgical treatments [13]. However, there is a lack of research on whether patients over 75 years old achieve the same level of clinical outcomes from ASD surgery as younger patients.

In this study, we aimed to investigate whether surgery for ASD in patients aged 75 and older is as effective in terms of clinical outcomes such as pain, disability, and health-related quality of life (HRQOL) improvement, as it is in younger patients. Additionally, this study sought to explore the rationale behind performing ASD surgery in patients over 75, despite the higher risks of morbidity and mortality.

## 2. Materials and Methods

### 2.1. Study Design and Patients

This retrospective review of prospectively collected data for ASD patients was approved by the Institutional Review Board of our hospital. A total of 151 patients who underwent ASD surgery between August 2014 and September 2020 were included in the study. The inclusion criteria were as follows: (1) Cobb angle >25° or sagittal vertical axis (SVA) >5 cm, pelvic tilt (PT) >25°, and pelvic incidence (PI)—lumbar lordosis (LL) >20°; (2) availability of one-year of follow-up data; and (3) diagnosis of ASD with sagittal imbalance and a treatment plan including corrective surgery. The exclusion criteria were as follows: (1) presence of other spinal diseases such as thoracic or cervical myelopathy; (2) severe pain in the knee, hip, or ankle joints that impedes walking; (3) peripheral vascular disease; (4) any syndromic, neuromuscular disease; (5) any serious uncontrolled medical comorbidity, such as sepsis or cancer that would cause disability; and (6) inability to answer the questionnaires on HRQOL.

### 2.2. Group Allocation

Patients were divided into two groups based on age. Among the total 151 patients, 93 patients were aged under 75 years and 58 patients were aged 75 years or older.

### 2.3. Surgical Procedures

All surgeries were performed by a single surgeon. Patients were positioned prone on a Jackson spine table for maximal lumbar lordosis, and simple radiographs were taken. Pelvic incidence-lumbar lordosis (PI-LL) was calculated using the radiographs to determine the desired correction angle and whether three-column osteotomy or posterior column osteotomy (PCO) would be suitable for the patient. Muscle dissection was performed from the upper instrumented vertebra-2 level and above to preserve ligaments and facet capsules. The long construct was sustained and degeneration of L5/S1 was avoided by fusion down to the sacrum and iliac screw insertion. Interbody fusion, compression between screws, cantilevering of rods, and other surgical techniques were applied to complement deformity correction. A thoraco–lumbo–sacral orthosis was applied to patients postoperatively for 3 months.

### 2.4. Surgical Outcome Measurements

The surgical outcomes were evaluated preoperatively and postoperatively using the visual analog scale (VAS) for low back pain, the Oswestry disability index (ODI), and the EuroQOL (EQ-5D). Radiological parameters were measured including SVA, PT, and PI-LL. The VAS is a 10 cm linear scale used to assess the degree of back pain with responses ranging from “no pain” and “most severe pain” on the left and right end of the scale, respectively. The ODI is a self-reporting questionnaire that measures back-related functional ability. It is composed of 10 questions, each with a 0–5 response scale range. The total score is calculated by adding all the scores of each item with a maximum score being 100. The EQ-5D is a scale to evaluate quality of life in five dimensions: mobility, self-care, usual activities, pain/discomfort, and anxiety/depression. Scores range from 0 to 1.00 with 0 meaning death and 1.00 meaning full health. The data were collected 3, 6, and 12 months after surgery.

### 2.5. Minimally Clinically Important Difference

The minimal clinically important difference (MCID) is a minimum change that is clinically meaningful to a patient. The MCID is at least 1.2 for the VAS, 11 points for the ODI, and 0.18 for the EQ-5D [18,19,20].

### 2.6. Perioperative Complications

Perioperative complications were defined as any event deviating from a normal postoperative course within 30 days after surgery. Perioperative complications were divided into medical complications and operative complications. Medical complications in this study included cardiac events, stroke/hemorrhage, pneumonia, pulmonary edema, thromboembolism, deep vein thrombosis (DVT), hepatitis, acute kidney injury (AKI), and sudden death. Among many operative complications, we chose to assess the most commonly occurring operative complications after ASD surgery, which are proximal junctional kyphosis (PJK) and proximal junctional failure (PJF).

### 2.7. Statistical Analysis

VAS, ODI, EQ-5D, and radiological parameters 12 months after the surgery were used as postoperative data. A Student *t*-test was used to compare continuous data, while a chi-square test was used to compare categorical variables. Improvements over time within each group were assessed using a repeated measure ANOVA. Comparison of VAS, ODI, EQ-5D, and radiological parameters across the two age groups was performed using an independent *t*-test, and an ANCOVA was used to adjust preoperative values affecting postoperative values. MCID and complication rates were assessed using a chi-square test. All statistical analyses were conducted using IBM SPSS statistics 26 (IBM Corp., Armonk, NY, USA), and the level of significance was set at *p* < 0.05 for all analyses.

## 3. Results

### 3.1. Descriptive Analysis

Complete surgical outcome data including VAS for lower back pain, ODI, and EQ-5D data were available for both groups 12 months after surgery. Sex distribution and BMI were similar in the two groups (*p* = 0.186 and *p* = 0.905, respectively). The BMD of the trochanter (*p* = 0.03) tended to be lower in the older group, while the BMD of the neck (*p* = 0.07) and BMD of the femur (*p* = 0.072) were not significantly different. The preoperative VAS for lower back pain, ODI, and EQ-5D scores were not significantly different in the older and younger age groups (*p* = 0.186, *p* = 0.083, and *p* = 0.231, respectively). There were no significant differences in preoperative spinopelvic parameters either, as all the *p* values were larger than 0.05. Only six patients scored ≥ 3 on the Charlson comorbidity index (CCI) in the entire patient group, and four of them were 75 years or older. There were no significant differences between the age groups in terms of preoperative comorbidities (*p* = 0.146) (Table 1).

The extent of instrumentation was assessed based on two characteristics: fusion length and iliac screw insertion (Table 1).

### 3.2. Surgical Outcome Analysis

The primary outcome—VAS and EQ-5D had no statistically valid *p* values when ANCOVA was performed. The ODI score with adjusted preoperative value also did not differ significantly between the younger and older patients 12 months after surgery (*p* = 0.087). No significant differences were found between the two groups in terms of the VAS for back pain and EQ-5D (*p* = 0.459 and *p* = 0.055, respectively) at 12 months after surgery (Table 2). The ODI (mean ± standard deviation) was 37.7 ± 17.0 in the younger group and 43.4 ± 17.3 in the older group 12 months after surgery. Without adjustment, the ODI was significantly different in the younger and older patients 12 months after surgery (*p* = 0.048).

**Figure 1:**
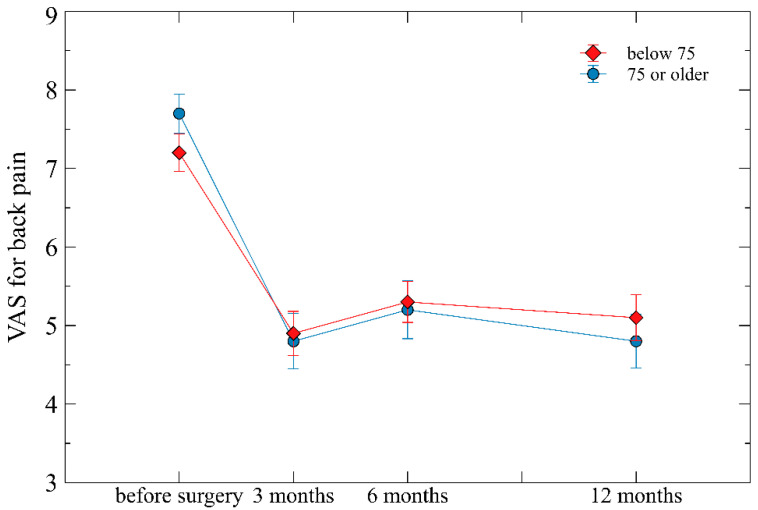
The overall change of visual analog scale (VAS) of back pain at follow-up assessments (preoperative, postoperative 3 months, 6 months, and 12 months) according to the age groups of below 75 years and over 75 years. Mean ± standard errors are plotted on the graph.

**Figure 2:**
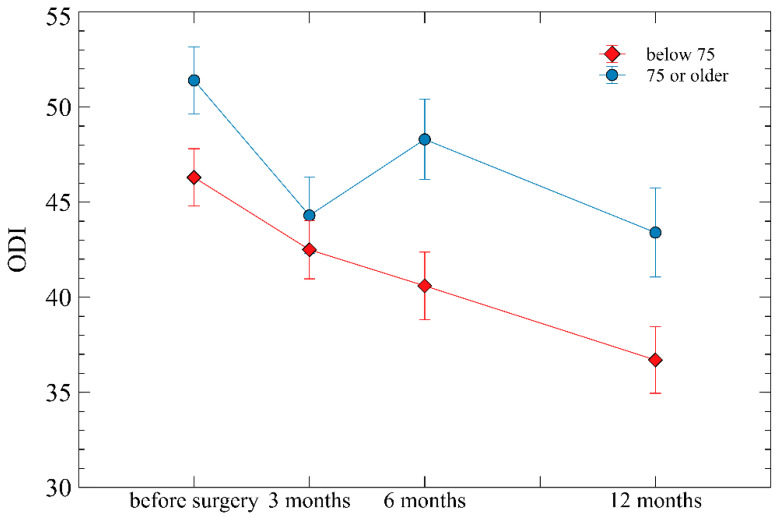
The overall change of Oswestry disability index (ODI) at follow-up assessments (preoperative, post-operative 3 months, 6 months, and 12 months) according to the age groups of below 75 years and over 75 years. Mean ± standard errors are plotted on the graph.

**Figure 3:**
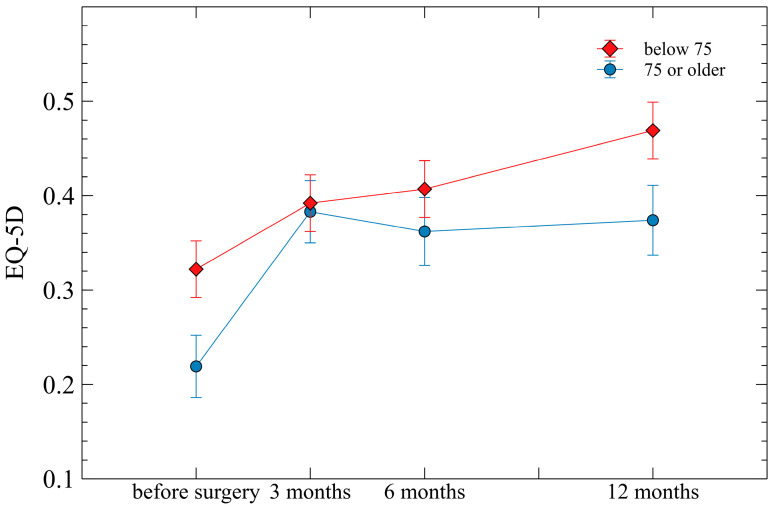
The overall change of EuroQOL (EQ-5D) at follow-up assessments (preoperative, postoperative 3 months, 6 months, and 12 months) according to the age groups of below 75 years and over 75 years. Mean ± standard errors are plotted on the graph.

Repeated measure ANOVA showed prominent changes over time for all three of VAS, ODI, and EQ-5D (all *p* < 0.001), suggesting that these variables improved markedly with time after surgery. The interactions between age group and time since surgery in terms of VAS for back pain, ODI, and EQ-5D scores were not significantly different (*p* = 0.301, *p* = 0.236, and *p* = 0.367, respectively) (Figure 1, Figure 2 and Figure 3).

For VAS for low back pain, 90 patients (59.6%) reached the MCID. Among them, 39 patients were 75 years and older and 51 patients were under 75 years, accounting for 67.2% and 53.8% of each group, respectively. For ODI, 71 patients (47.0%) reached the MCID with 23 of them aged 75 years and older, accounting for 46.6% of the elderly group. In the younger group, the proportion of patients who reached the MCID was greater at 52.7%. There were no significant correlations between the age group and the likelihood of reaching the MCID (VAS: *p* = 0.131, ODI: *p* = 0.152) (Table 3).

Five patients in the total group experienced medical complications, three in the younger group and two in the older group. The medical complication rate did not differ between the two groups. On the other hand, correlations were found between age group and operative complications. PJK occurred in 48.3% of patients from the older age group and only 20.4% of patients in the younger group (*p* < 0.001). Similarly, 37.9% of patients in the older group developed PJF while only 15.1% of patients in the younger age group did (*p* = 0.001) (Table 4).

### 3.3. Radiological Outcome Analysis

There were no significant differences in preoperative radiological parameters between the two groups (PT: *p* = 0.699; PI-LL: *p* = 0.573; and SVA: *p* = 0.647) and after 12 months of surgery (*p* = 0.675, *p* = 0.948, and *p* = 0.108, respectively) (Table 5).

Repeated measure ANOVA showed no significant interactions between age group and follow-up time for all parameters (*p* = 0.907, *p* = 0.492, and *p* = 0.289 for PT, PI-LL, and SVA, respectively). Moreover, there were significant improvements in PT, PI-LL, and SVA 12 months after surgery compared to preoperative data (all *p* < 0.001).

## 4. Discussion

The objective of this study was to determine whether the age of 75 can serve as a prognostic factor for predicting the outcomes of ASD surgery. Preoperative, postoperative 3-month, 6-month, and 12-month VAS, ODI, and EQ-5D scores, other radiological parameters, and complications were compared between the younger and the older age groups. Contrary to our initial expectations, we could not demonstrate a crucial association between old age and surgical outcome, suggesting that ASD surgery can be performed on elderly patients even older than 75 years without compromising pain, instability, or health-related quality of life.

The 12-month post-operative clinical parameters were statistically insignificant between the two groups. One possible reason is the small number of elderly patients with serious comorbidities in the study. The ratio of patients with a CCI of 3 or higher was similar in the two groups (*p* = 0.146), accounting for only 2–5% of each group. The other possible reason lies in the surgical approach. Generally, the posterior-only approach and PCO are known to show lower rates of complications [21]. All our patients underwent posterior-only approaches, and the ratio of patients who received PCO was higher in the elderly group (*p* = 0.011). Daubs et al. showed that ODI scores did not differ significantly between patients older than 69 and under 69, which agrees with our results [22]. In addition, all three clinical parameters showed a noticeable increase in both groups (all *p* < 0.001). These results suggest that patients over 75 should enjoy equal benefit to patients aged under 75 in terms of function, pain, and quality of life.

The prominent post-operative improvements in all parameters found in our study were consistent with the improvements reported in the study by Sciubba et al. which showed significant improvements in patient-reported outcomes in patients over 75 after 2 years following ASD surgery [13]. Karabulut et al. conducted a similar study on patients over 70 years who underwent ASD surgery and drew the same conclusion [12]. In the literature review by Drazin et al., the VAS and ODI scores of patients over 60 years improved significantly after ASD surgery with a mean score decrease of 5.2 points in VAS scores and 24.1 in ODI [23]. The novelty of our study is that, compared to the papers mentioned above which were single-arm cohort studies on elderly patients only, our study was a cohort study comparing two age groups.

In terms of VAS, both groups showed similar trends up to 12 months. However, for EQ-5D and ODI, as time passed after surgery, the older age group exhibited more unfavorable scores compared to the young age group, and the gap between the two groups also increased (Figure 1, Figure 2 and Figure 3). This is considered because VAS evaluates only pain itself and is independent of age, whereas EQ-5D and ODI include age-dependent factors in their evaluation.

Previous research has reported that with increasing age, poorer HRQOL is observed [24]. Specifically, in our study, patients over the age of 75, who were set as the cut-off, are more likely to have comorbidities compared to younger patients, as well as issues with joints other than the spine and internal medical problems. Considering these factors, the severity of HRQOL can widen over time in comparison to younger patients.

In the elderly over 75, there is a higher likelihood of disability not just from spine issues but also from other causes. Particularly in the case of ODI, it evaluates age-dependent factors such as personal care (washing, dressing), lifting, sleeping, sex life, and traveling. Considering these, the extent of disability is likely to be higher over time compared to younger patients. This trend is also evident in the research of Michael H. Weber et al. Their study also showed that with time, older age groups exhibited higher ODI scores, and the gap between them and younger patients increased [25].

In terms of the MICD in VAS and ODI, we did not find significant relationships between the number of patients reaching the MCID and age. Khan et al. conducted a study on 850 patients who had undergone posterolateral lumbar spinal fusion and compared the radiological and clinical outcomes in a younger group (18–54 years), a middle-aged group (55–69 years), and a senior age group (70 years or older) [26]. No significant differences were found among the age groups in terms of the proportion of patients satisfying the MCID for VAS and ODI scores, which was consistent with our results.

There were conflicting results in terms of medical and operative complications. Only operative complications such as PJK and PJF were meaningfully correlated with age. Patients who were 75 years or over were 3.6 times, and 3.4 times more likely to have PJK and PJF, respectively. Bridwell et al. and Kim et al. suggested that older age was a risk factor for PJK, which is consistent with our results [27,28]. Medical complications did not show such a correlation with age. In a study conducted on patients over 60 years, Daubs et al. [22] reported that patients older than 69 years were nine times more likely to develop medical complications than those aged less than 69 years. The different surgical approaches might have influenced these conflicting results. Only half of the patients in Daubs et al.’s studies underwent a posterior-only approach, while in our study, all patients underwent a posterior-only approach. Also, the mean operation time in Daubs et al.’s study (605 min) was much longer than that in our study (442 min) in the older group [22].

Interestingly, most of the radiological parameters were not significantly different between the two groups, both preoperatively and postoperatively. This implies that patients over 75 years old can expect equivalent radiological outcomes to those of younger patients following surgery. This is also supported by the retrospective cohort study performed by Khan et al. which showed no significant differences in postoperative radiological parameters including PT, PI, LL, and PI-LL among three age groups: 18–54 years, 55–69 years, and 70 years and older [26]. Bridwell et al. reported that radiological parameters improved after ASD surgery, but they did not change over the entire follow-up period [29]. In contrast, in our research, SVA increased significantly with time in both groups. This can be explained by the fact that patients in Bridwell et al.’s study had a broad range of ages, while our participants were predominantly elderly. In Bridwell et al.’s study, only 24.8% were over 60 years old, while 90.1% of our patients were distributed in this age group. In general, SVA increases with age, and this accounts for the conflicting findings between our study and that of Bridwell et al. [27,30].

Indeed, there were some limitations to our study. The first limitation was its retrospective design, meaning that critical data may have been missing, thereby affecting the results. Second, the size of the sample was relatively small, as the data were collected from only one surgical center, and the homogeneity of our data could be an obstacle in applying our conclusions to other races. Further studies with larger sample sizes and a broader range of races are needed to facilitate wider generalization of the results. Third, the MCID threshold values we chose might not have been the most suitable for our patient group as these values were extracted from literature reviews conducted on Western populations. Arima et al. suggested that the Japanese floor-based lifestyle was the reason that the Japanese patients experienced a lesser awareness of treatment improvements. Since our study population shared a similar cultural background to that of Japan, the results might differ when an MCID threshold specific to Asian populations is used.

## 5. Conclusions

There were no significant differences between the two groups in pain, disability, and health-related quality of life, all radiological parameters were assessed. Rates of the MCID and medical complications did not differ statistically according to age, while PJF and PJK did. These results imply that ASD surgery is not associated with poorer post-operative outcomes in patients aged 75 years or more compared to those less than 75 years. We concluded that an age of 75 years cannot be used as a cutoff for the prediction of poor prognosis following ASD surgery. However, the higher risk of operative complications and the necessity for revision surgery in ASD patients 75 years or older should still be considered carefully.

## Figures and Tables

**Table 1:** Sample Descriptive (n = 151).

	Below 75 (n = 93)	75 Years and Older (n = 58)	*p* Value
Age (yrs)	66.4 ± 6.8	77.8 ± 2.4	**<0.01**
Female (n [%])	74 (79.6%)	51 (87.9%)	0.186
BMI (kg/m^2^)	25.7 ± 4.1	25.7 ± 3.1	0.905
BMD			
Neck	0.648 ± 0.114	0.613 ± 0.114	0.07
Trochanter	0.576 ± 0.109	0.222 ± 0.256	**0.03**
Femur	0.775 ± 0.132	0.735 ± 0.132	0.072
Preoperative clinical outcome variables			
VAS for back pain	7.2 ± 2.3	7.7 ± 1.9	0.231
ODI	46.9 ± 14.6	50.0 ± 13.2	0.186
EQ-5D	0.304 ± 0.297	0.222 ± 0.256	0.083
Preoperative radiological parameters			
PT (°)	32.0 ± 13.5	32.9 ± 13.0	0.699
PI-LL (°)	50.8 ± 29.6	48.2 ± 24.2	0.573
SVA (cm)	15.55 ± 8.87	16.21 ± 7.88	0.647
No. of comorbidities			
0 (n [%])	38 (40.9%)	16 (27.6%)	0.277
1 (n [%])	30 (32.3%)	21 (36.2%)	
2 (n [%])	20 (21.5%)	14 (24.1%)	
3 (n [%])	3 (3.2%)	6 (10.3%)	
4 (n [%])	2 (2.2%)	1 (1.7%)	
CCI ≥ 3	2 (2.2%)	4 (6.9%)	0.146
Extent of instrumentation			
fusion length (levels)	7.85 ± 1.7	7.60 ± 1.0	0.336
iliac screw insertion (n [%])	56 (65.1%)	41 (74.5%)	0.239
Osteotomy			
PCO (n [%])	32 (37.2%)	32 (59.3%)	**0.011**
Operation			
Operation time (min)	446 ± 85	442 ± 89	0.751

Values are mean ± standard deviation. Bold values indicate statistically significant (*p* < 0.05). BMI indicates body mass index; BMD, bone mineral density; VAS, visual analog scale; ODI, Oswestry disability index; EQ-5D, EuroQOL; PT, pelvic tilt; PI, pelvic incidence; LL, lumbar lordosis; SVA, sagittal vertical axis; PCO, posterior column osteotomy.

**Table 2:** Postoperative Clinical Outcome Variables.

		Below 75	75 Years and Older	*p* Value
VAS for back pain	PREOP	7.2 ± 2.3	7.7 ± 1.9	0.231
	POD #3M	4.8 ± 2.8	4.8 ± 2.7	0.894 (0.860)
	POD #6M	5.3 ± 2.8	5.2 ± 2.8	0.972 (0.695)
	POD #12M	5.1 ± 2.6	4.8 ± 2.6	0.459 (0.342)
ODI	PREOP	46.9 ± 14.6	50.0 ± 13.2	0.186
	POD #3M	42.8 ± 14.9	43.2 ± 16.1	0.871 (0.660)
	POD #6M	40.7 ± 16.7	47.4 ± 17.7	**0.030 (0.035)**
	POD #12M	37.7 ± 17.0	43.4 ± 17.3	0.048 (0.087)
EQ-5D	PREOP	0.304 ± 0.297	0.222 ± 0.256	0.083
	POD #3M	0.379 ± 0.282	0.390 ± 0.255	0.826 (0.886)
	POD #6M	0.411 ± 0.297	0.361 ± 0.297	0.233 (0.254)
	POD #12M	0.457 ± 0.269	0.368 ± 0.288	0.055 (0.125)

Values are mean ± standard deviation. Bold values indicate statistically significant (*p* < 0.05). Parentheses means baseline adjusted *p*-value results from ANCOVA. VAS, visual analog scale; ODI, Oswestry disability index; EQ-5D, EuroQOL.

**Table 3:** Minimum Clinically Important Differences.

		≥MCID	<MCID	*p* Value
VAS	Below 75	50 (53.8%)	43 (46.2%)	0.131
	75 yrs and older	39 (67.2%)	19 (32.8%)	
ODI	Below 75	49 (52.7%)	44 (47.3%)	0.152
	75 yrs and older	27 (46.6%)	31 (53.4%)	

VAS, visual analog scale; ODI, Oswestry Disability Index.

**Table 4:** Numbers of Patients Who Went Through Postoperative Complication.

	Below 75 (n = 93)	75 Years and Older (n = 58)	*p* Value
Medical complication (n)			
Cardiac event	0	0	
Stroke/hemorrhage	1	0	
Pneumonia	0	0	
Pulmonary edema	1	2	
Thromboembolism	1	0	
DVT	0	0	
Hepatitis	0	0	
AKI	0	0	
Sudden death	0	0	
Total	3	2	0.638
Operative complication (n [%])			
PJK	19 (20.4)	28 (48.3)	**<0.001**
PJF	14 (15.1)	22 (37.9)	**0.001**
Revision surgery (n [%])	6 (6.5%)	5 (8.6%)	0.422

Bold values indicate statistically significant (*p* < 0.05). DVT indicates deep vein thrombosis; AKI, acute kidney injury; PJK, proximal junctional kyphosis; PJF, proximal junctional failure.

**Table 5:** Radiological Parameters.

		Below 75	75 Years and Older	*p* Value
PT (°)	PREOP	32.0 ± 13.5	32.9 ± 13.0	0.699
	POD #12M	24.8 ± 12.7	25.7 ± 12.5	0.675
PI-LL (°)	PREOP	50.8 ± 29.6	48.2 ± 24.2	0.573
	POD #12M	18.1 ± 20.6	17.9 ± 14.8	0.948
SVA (cm)	PREOP	15.55 ± 8.87	16.21 ± 7.88	0.647
	POD #12M	7.46 ± 5.93	9.05 ± 4.7	0.108

Values are mean ± standard deviation. PT, pelvic tilt; PI, pelvic incidence; LL, lumbar lordosis; SVA, sagittal vertical axis.

## Data Availability

All data included in this study can be provided by contacting oshjkim@gmail.com.
